# Sex determination in the GIFT strain of tilapia is controlled by a locus in linkage group 23

**DOI:** 10.1186/s12863-020-00853-3

**Published:** 2020-04-29

**Authors:** Khanam Taslima, Stefanie Wehner, John B. Taggart, Hugues de Verdal, John A. H. Benzie, Michaël Bekaert, Brendan J. McAndrew, David J. Penman

**Affiliations:** 1 Institute of Aquaculture, Faculty of Natural Sciences, University of Stirling, Stirling, Scotland UK; 2 Department of Fisheries Biology and Genetics, Bangladesh Agricultural University, Mymensingh, 2202 Bangladesh; 3 WorldFish Centre, Jalan Batu Maung, Bayan Lepas, Penang, Malaysia; 4 CIRAD, UMR ISEM, F-34398 Montpellier, France; ISEM, Univ Montpellier, CNRS, EPHE, IRD, Montpellier, France; 5 School of Biological Earth and Environmental Sciences, University College Cork, Cork, Ireland

**Keywords:** Sex determination, GIFT, BSA-ddRADseq, MAS, Sex-ratio control

## Abstract

**Background:**

Tilapias (Family Cichlidae) are the second most important group of aquaculture species in the world. They have been the subject of much research on sex determination due to problems caused by early maturation in culture and their complex sex-determining systems. Different sex-determining loci (linkage group 1, 20 and 23) have been detected in various tilapia stocks. The ‘genetically improved farmed tilapia’ (GIFT) stock, founded from multiple Nile tilapia (*Oreochromis niloticus*) populations, with some likely to have been introgressed with *O. mossambicus*, is a key resource for tilapia aquaculture. The sex-determining mechanism in the GIFT stock was unknown, but potentially complicated due to its multiple origins.

**Results:**

A bulk segregant analysis (BSA) version of double-digest restriction-site associated DNA sequencing (BSA-ddRADseq) was developed and used to detect and position sex-linked single nucleotide polymorphism (SNP) markers in 19 families from the GIFT strain breeding nucleus and two Stirling families as controls (a single XY locus had been previously mapped to LG1 in the latter). About 1500 SNPs per family were detected across the genome. Phenotypic sex in Stirling families showed strong association with LG1, whereas only SNPs located in LG23 showed clear association with sex in the majority of the GIFT families. No other genomic regions linked to sex determination were apparent. This region was validated using a series of LG23-specific DNA markers (five SNPs with highest association to sex from this study, the LG23 sex-associated microsatellite *UNH898* and *ARO172,* and the recently isolated *amhy* marker for individual fish (*n* = 284).

**Conclusions:**

Perhaps surprisingly given its multiple origins, sex determination in the GIFT strain breeding nucleus was associated only with a locus in LG23. BSA-ddRADseq allowed cost-effective analysis of multiple families, strengthening this conclusion. This technique has potential to be applied to other complex traits. The sex-linked SNP markers identified will be useful for potential marker-assisted selection (MAS) to control sex-ratio in GIFT tilapia to suppress unwanted reproduction during growout.

## Background

The mechanisms underlying sex determination show considerable variation in vertebrates. Nearly all mammals have a male heterogametic sex-determining system (XX/XY) with the Y-linked *Sry* gene regulating sex determination [1, 2]. In contrast, female heterogamety (WZ/ZZ) occurs in birds and some snakes, where the Z-linked *Dmrt1* gene triggers male sex development by a double dosage mechanism [3, 4]. Simple (male or female heterogametic) to complex (polygenic) genetic sex determination, environmental sex determination and sometimes interaction between genes and environmental factors have been observed in fish, lizards, turtles and amphibians [3].

Fish are an extremely diverse group of organisms, with the underlying mechanisms of sex determination not being strongly conserved among taxa. These can vary among closely related species, and even show intraspecific variation. For example, three different genes responsible for sex determination have been identified in three different fish species in one genus: *Dmy/Dmrt1by* in *Oryzias latipes* [5, 6], *Sox3y* in *O. dancena* [7] and *Gsdfy* in *O. luzonensis* [8]. Different components of the transforming growth factor beta (TGF-β) have been identified as strong candidates for master sex-determining genes in different fish species - *Amhy* in *Odontesthes hatcheri* [9], *Amhr2* in *Takifugu rubripes* [10] and *Amhy* in the Nile tilapia, *Oreochromis niloticus* [11, 12]. A sexually dimorphic immune-related gene only present on the Y chromosome (*sdY*) is the master sex-determining gene in the rainbow trout, *Oncorhynchus mykiss* [13] and this male-specific gene have been found to be conserved across the salmonids [14].

Tilapia show great species diversity, with more than 70 freshwater species and a few brackish water species being found in Africa and the Middle East. The Nile tilapia plays a significant global role in commercial aquaculture production. Following the early introductions of Nile tilapia to different Asian countries, the genetic quality of these stocks often deteriorated, probably because of genetic founder and bottleneck effects followed by inbreeding depression, owing to the import of limited numbers of fish from Africa and low effective population sizes [15]. In addition to these, the purity of Nile tilapia aquaculture stocks has deteriorated due to introgression with the less desirable Mozambique tilapia, *O. mossambicus*, introduced to Asia before *O. niloticus* for aquaculture and now feral in many countries [16, 17]. To improve the genetic quality of farmed stocks of this species, and more generally to demonstrate the potential for genetic improvement in warm water aquaculture, the genetically improved farmed tilapia (GIFT) strain was developed by the WorldFish Centre through selective breeding. The GIFT base population was developed from multiple wild (African) and domesticated (Asian) populations of Nile tilapia [15, 18] and has made a significant contribution to world tilapia aquaculture production. Its success has led to many other selective breeding programmes being developed [19].

Both male (XX/XY) and female (WZ/ZZ) heterogametic sex-determining systems and environmental influences on sex are evident in different tilapia species. The variety of sex-determining systems in tilapia and the demand for single sex (monosex male) culture (to avoid unwanted reproduction and to take advantage of faster growth in males) have encouraged researchers to elucidate sex determination in tilapia. Different sex-determining loci have been mapped in different chromosomes (linkage groups, LGs) in tilapia species. From microsatellite marker-based studies, loci in LG1 and LG3 have been associated with phenotypic sex in blue tilapia, *O. aureus,* which possesses primarily female heterogametic sex determination [20], whereas a male heterogametic sex determination locus was found in LG1 in Mozambique tilapia originating from South Africa [21].

Nile tilapia shows male heterogamety (XX/XY) which may sometimes interact with minor genetic or environmental factors to result in the phenotypic sex [22]. Two different XX/XY sex-determining loci (in LG1 and LG23) have been mapped in different stocks of Nile tilapia. One locus was mapped to LG1 in the Stirling strain of Nile tilapia, originally derived from Lake Manzala in Egypt, using BSA-mediated microsatellite marker analysis [23] and restriction-site associated DNA sequencing (RADseq) [24]. Thermosensitivity associated with loci in LG20 [25], and LG1, LG3 and LG23 [26, 27] has also been observed in Stirling Nile tilapia.

In a stock in Israel, derived from the Swansea stock of Nile tilapia (itself derived from the Stirling stock), another XX/XY sex-determining locus, in LG23, was found using simple sequence repeats (SSR) and sex-specific markers analysis [28, 29]. A tandemly-duplicated variant of the *Amh* gene, *Amhy* (associated with male sex determination), was identified as a candidate sex determiner in this stock [11]. The same *Amh* variant was identified in a Japanese strain of Nile tilapia, originating from Egypt, which the authors named *AmhΔy* to distinguish it from another tandemly duplicated copy of the *Amh* gene, which they called *Amhy* due to its Y-specific expression and other experimental evidence from knocking out the gene in XY individuals and gene transfer into XX individuals [12]. *Amhy* is located immediately downstream of *AmhΔy* in the Y haplotype in LG23 and the coding sequence is identical to the X-linked *Amh* except for a 5608 bp promoter deletion and a single base substitution identified in exon II (the latter thought to have a critical role in male sex determination).

In recent years, genotyping-by-sequencing (GBS) approaches based on RADseq [30, 31] have allowed rapid and cost-efficient mapping of sex determination in a range of fish species (e.g. Nile tilapia *O. niloticus* [24]; zebrafish *Danio rerio* [32]; European seabass *Dicentrarchus labrax* [33]), even in species with little or no existing genomic resources (e.g. Atlantic halibut *Hippoglossus hippoglossus* [34]; hāpuku *Polyprion oxygeneios* [35]). Such work has generally been based on detailed analysis of one or a few families, followed by verification of sex-linked markers in unrelated individuals. Given the evidence for intraspecific and interspecific variation in sex determination in tilapia, and the synthetic base population from which GIFT was developed, it was desirable to develop a novel approach to allow relatively rapid screening of sex determination in multiple families in the GIFT strain.

In BSA-based gene mapping studies, samples are pooled based on the phenotypic differences for a particular trait of interest and the genetic analysis then explores differences between the pools [36]. It has been previously used in mutation detection and disease studies in humans [37, 38] and genetic linkage studies in plants [39–41]. Subsequently the BSA approach has been combined with different molecular marker technologies to identify quantitative trait locus (QTL) associated with disease resistance and sex-related markers in different fish species [23, 42–45]. Molecular marker development and genotyping needed to be performed separately for most earlier BSA-based marker analyses, which is costly and time-consuming. Combining BSA with simultaneous marker discovery and genotyping afforded by GBS should allow multiple families to be analysed in a single sequencing library in a rapid and cost-effective manner. A BSA-ddRASDseq approach was taken to explore the sex-determining mechanisms operating in the GIFT tilapia strain and validated by additional analysis of other informative sex-linked markers.

## Results

### Confirmation of LG1 sex association in Stirling families

The phenotypic sex-ratios in the two Stirling families were not significantly different from the expected 1:1 ratio (Additional file 2: Table S1). SNP markers (*Oni23063* and *Oni28137* [24]) in LG1 showed strong, significant association with sex for both of the families (*p <* 0.001 for both SNP markers), as did the LG1 microsatellite marker (*UNH995, p <* 0.001 for both families; Additional file 5: Data S1); similar results have also been reported in a previous publication on the same stock [24]. In contrast, there was no association of an LG20 (*Oni3161* [25]) marker with the phenotypic sex in either family. On this basis, these two Stirling Nile tilapia families were used as positive controls for the BSA-ddRADseq analysis.

### Generating BSA-ddRAD loci in Stirling and GIFT families

In total, 28,506,297 paired-end reads were generated from the two sequencing runs. As a result of the sample demultiplexing process, 83.6% of the paired-end reads were retained (Additional file 3: Table S2). Stacks analysis of the filtered reads identified between 9948 and 16,711 RAD loci per family (Additional file 3: Table S2). Of these, between 1432 to 3402 informative biallelic SNPs were identified per family and used for subsequent association analysis. The pooled samples were replicated four times in the first run while no replication was used in the second run. More reads were obtained per family in the first run, but the replication did not show any major difference with regard to the number of polymorphic filtered loci retrieved for further analysis (Additional file 3: Table S2).

### Mapping of sex-linked region from BSA-ddRADseq analysis

**Fig. 1:**
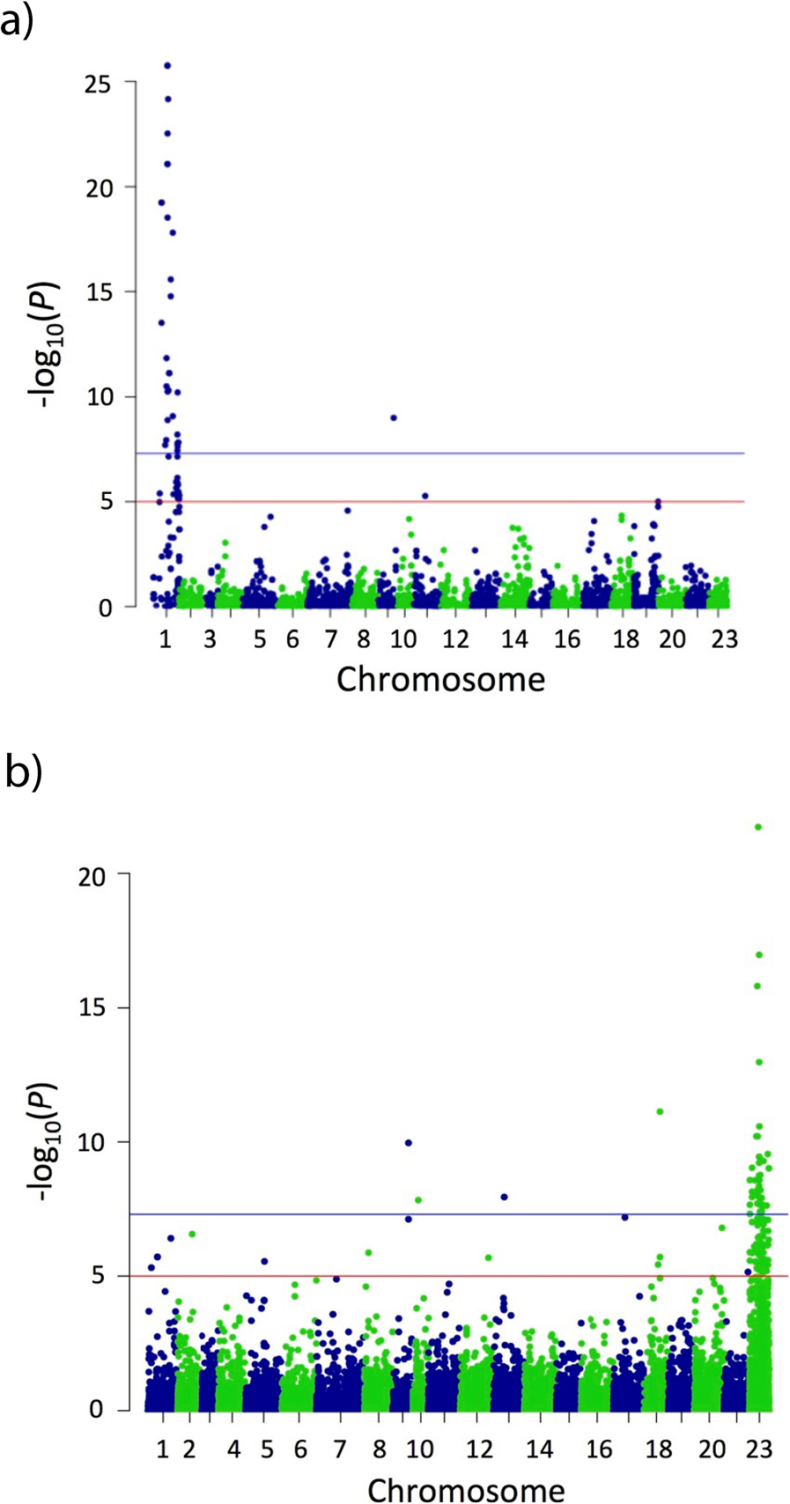
Genome-wide association plots, from combined families of Stirling (**a**) and GIFT (**b**). Each dot represents a SNP and the Y-axis represents the magnitude of association (−log_10_*P* value of F-test) of the SNP with phenotypic sex, while the X-axis represents the position in the linkage groups of the assembled Nile tilapia genome. The alternating blue and green colours are used to distinguish between chromosomes. The red solid line represents a *q*-value of 0.05 and the blue solid line represents a *q*-value of 0.01 (adjusted to take multiple tests into account). **a** SNPs significantly associated with the phenotypic sex were identified in LG1 for Stirling families **b** SNPs in LG23 showed highest significant association with the phenotypic sex in GIFT families

For the Stirling families, SNP markers highly associated with phenotypic sex clustered in LG1, as expected (Fig. 1a). No markers from other LGs with high association with phenotypic sex were detected. BSA-ddRADseq based tilapia sex determination analysis thus confirmed the major sex-determining locus (LG1) in the two Stirling families [24].

SNPs with high association with phenotypic sex clustered only in LG23 in the GIFT families (Fig. 1b). No other significant associations appeared across the rest of the genome. A strong significant association was found in 12 GIFT families, while four families showed weaker but significant association, with some “noise” in the lower part of the graphs (Families 1, 2, 6 & 10; Additional file 1: Fig. S1) and three families did not show any significant association (Families 5, 14 & 19; Additional file 1: Fig. S1).

### Association analysis between genotype and phenotype at the family and population level, using individual marker assays

#### Sex-linked markers from the BSA-ddRADseq analysis

**Fig. 2:**
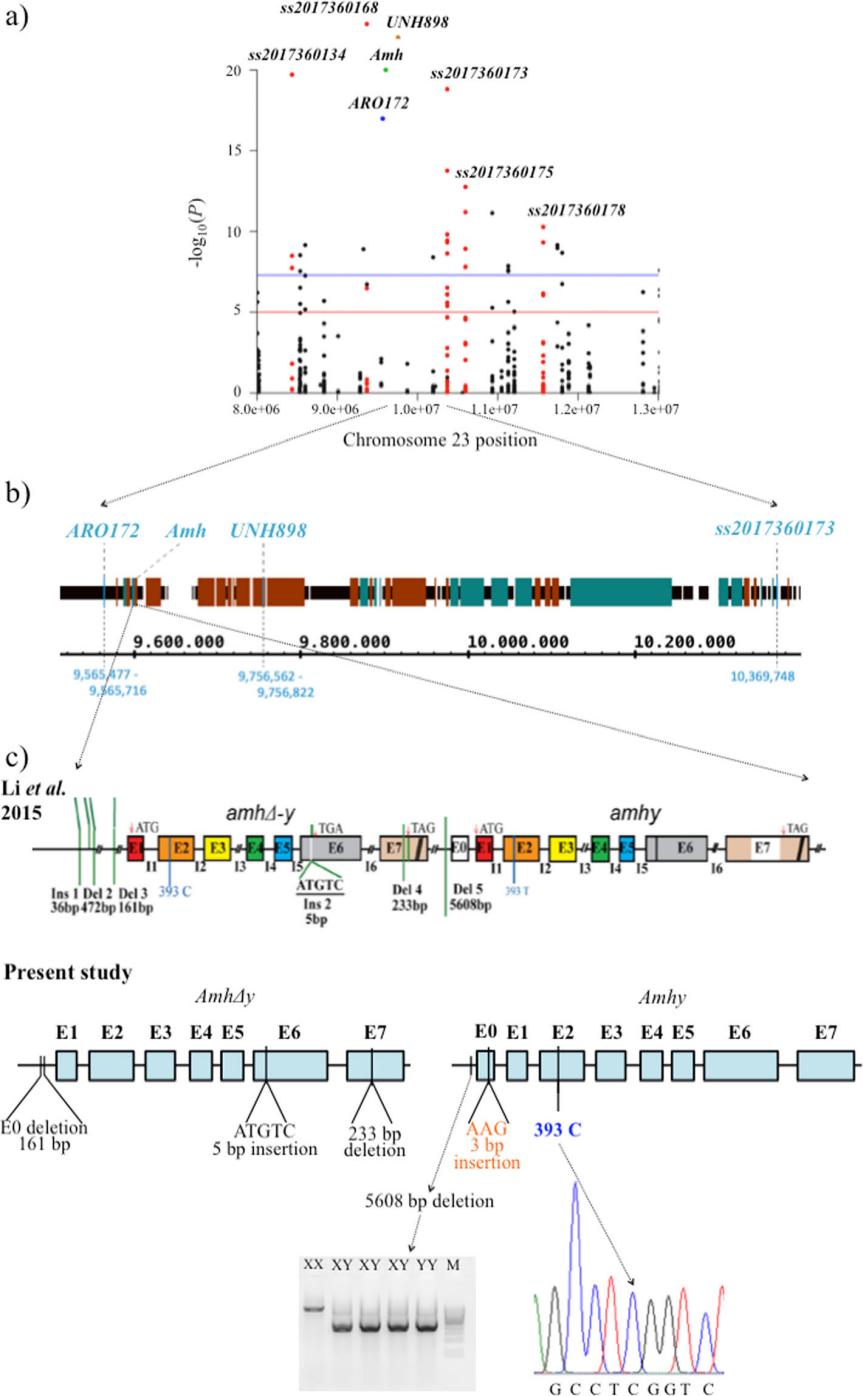
Detailed diagram of the putative XX/XY sex-determining region in LG23 in GIFT. **a** Position of the five sex-linked SNPs along the assembled Nile tilapia LG23. Each dot (red and black) represents the magnitude of association between the particular SNP and phenotypic sex for each family. SNPs with red dots were designed from BSA-ddRAD analysis for individual analysis of GIFT samples. Candidate sex-determining gene - *Amh* (green dot) including two sex-linked microsatellite markers (*UNH898* – yellow dot, *ARO172* – blue dot) are also located within this region (previous study, −log_10_*P* values were random). **b** Gene content information in the region of higher association (position 9,560,000 to 10,370,000). This includes 32 genes, of which 14 are annotated, with 26 gaps (19 to 29,961 nt; white regions). Green: genes on the plus strand, red: genes on the minus strand, black: normal nts with no identified gene. **c** Similarities and dissimilarities in the analysed Y-linked *AmhΔy* and *Amhy* between GIFT and the previous study of *Amh* gene [12]; *AmhΔy* in GIFT had exon 0 deletion (161 bp), 5 bp insertion in exon VI (ATGTC) and 233 bp deletion in exon VII compared to *Amh* (i.e. no differences were detected between the two studies); *Amhy* in GIFT had the 5608 bp promoter deletion previously observed in *Amhy* [12], but had a 3 bp insertion (AAG) in exon 0 and lacked the C → T substitution observed in exon II

KASP assays were developed for the five most significantly sex-associated SNPs from the BSA-ddRADseq analysis of the GIFT families and screened in six selected GIFT families, including one which did not show a significant association with sex (see Materials and Methods). The physical positions of these five SNPs were localised to a 3 Mb region of the Nile tilapia genome (Fig. 2a). All of these SNP markers were confirmed to be significantly associated with phenotypic sex in the GIFT families where the sire was heterozygous (informative) for the SNP (Table 1). However, none of the markers were fully diagnostic by themselves.

**Table 1:** Association analysis between phenotypic sex and five SNP markers derived from BSA-ddRADseq analysis, for 6 GIFT families and 50 GIFT broodstock. Values are probability of association (*p*). n.s. – not significant. n.a. - test not appropriate as both parents were homozygous

		*ss2017360134*	*ss2017360168*	*ss2017360173*	*ss2017360175*	*ss2017360178*
Family 1		n.a.	n.s.	n.a.	n.a.	n.a.
	Dam	C/C	C/C	T/T	A/A	G/G
	Sire	C/C	C/T	C/C	A/A	A/A
Family 2		< 0.01	< 0.01	< 0.01	n.a.	n.a.
	Dam	C/C	C/C	C/C	T/T	G/G
	Sire	C/T	C/T	C/T	A/A	G/G
Family 3		< 0.01	< 0.01	< 0.01	n.a.	n.s.
	Dam	C/C	C/C	C/C	A/A	A/G
	Sire	C/T	C/T	C/T	A/A	G/G
Family 4		n.a.	n.a.	< 0.01	n.s.	n.s.
	Dam	C/C	C/C	C/T	A/T	A/G
	Sire	C/C	C/C	C/T	A/A	A/A
Family 7		n.a.	n.a.	< 0.01	< 0.01	n.s.
	Dam	C/C	C/C	C/C	A/A	A/G
	Sire	C/C	C/C	C/T	A/T	G/G
Family 19		< 0.01	< 0.01	< 0.01	n.a.	< 0.01
	Dam	C/C	C/C	C/C	A/A	G/G
	Sire	C/T	C/T	C/T	A/A	A/G
Broodstock		n.s.	n.s.	< 0.01	< 0.01	n.s.

In the case of the broodstock alone (*n* = 50), two of the five SNP markers (*ss2017360173* and *ss2017360175*) showed significant association with the phenotypic sex (*p* < 0.01 in both cases, Table 1), but again were not fully diagnostic. Six of the males were homozygous and four of the females were heterozygous for the marker *ss2017360173* (Additional file 6: Data S2).

#### Markers associated with the Amh gene(s)

From the BSA-ddRADseq and SNP analysis described above, sex determination in the GIFT strain was strongly associated with the region in LG23 containing the *Amh* gene. This gene is reported to be a strong candidate for a sex-determining gene in this species [11, 12] (Fig. 2).

The allelic distributions of the two LG23 microsatellite markers known to be closely linked with *Amh* (*UNH898* and *ARO172*) were found to be significantly associated with the phenotypic sex when tested across all the families (*p* < 2.2 × 10^− 16^ for each marker) and each family separately for each marker (Additional file 4: Table S3). For the population data (50 broodstock), these two microsatellite markers were also highly associated with the phenotypic sex (*p*-value 7.62 × 10^− 6^ and 6.54 × 10^− 7^ respectively). The 267 allele for *UNH898* and 274 allele for *ARO172* marker were nearly always associated the male phenotype, irrespective of family and broodstock, with a few exceptions (see below) (Table 2 and Additional file 6: Data S2).

**Table 2:** Agreement and disagreement of phenotypic sex and genotype segregation for each family and broodstock studied for two microsatellite markers and markers in the variation of *Amh* gene (deletion in *Amh* exon VII and insertion in *Amh* exon VI) in Y chromosome

ID	Observed phenotype	Female expected genotype	Male expected genotype
Family 1	Female	19	1
	Male	5	15
Family 2	Female	19	1
	Male	7	13
Family 3	Female	18	2
	Male	2	18
Family 4	Female	20	0
	Male	0	20
Family 7	Female	15	0
	Male	0	15
Family 19	Female	16	4
	Male	4	16
Broodstock	Dam	23	1
	Sire	3	23

**Fig. 3:**
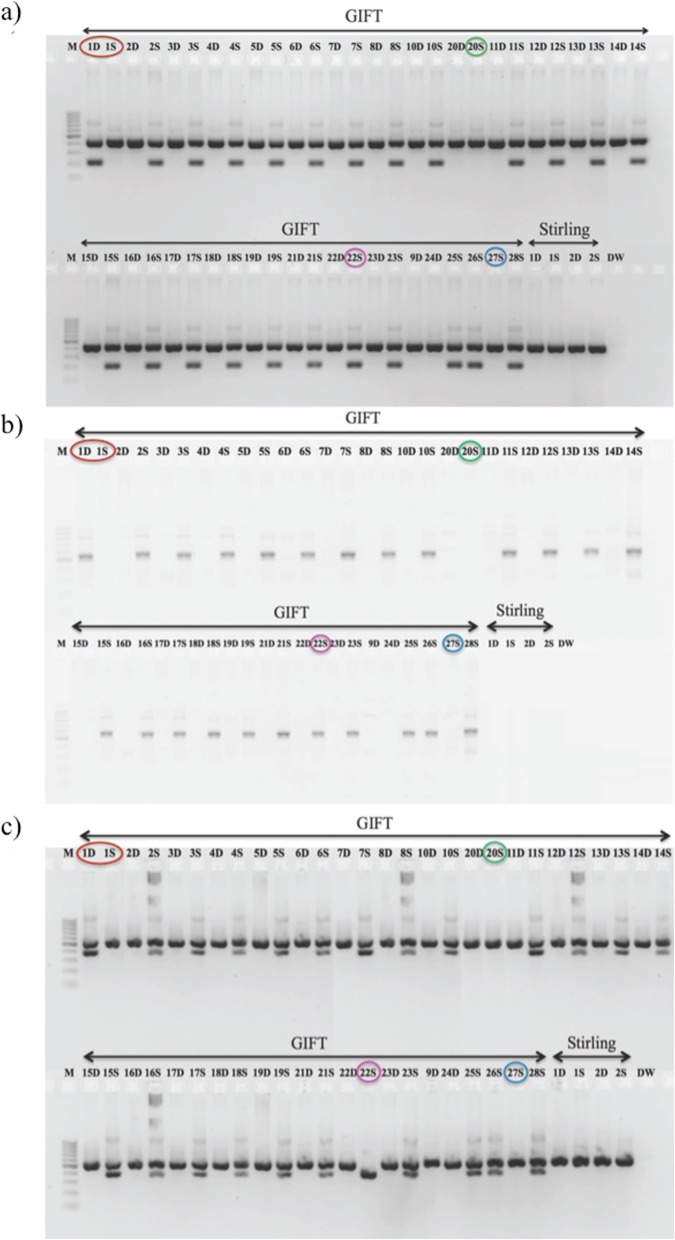
Amplified PCR products on 1.5% agarose gel using markers from the different regions in the variations of the *Amh* gene for 50 GIFT and 4 Stirling broodstock. **a** Deletion in *Amh* exon VII (*AmhΔy* [12]). A 439 bp band was evident in all individuals and an extra 206 bp band was present in nearly all males. **b** Insertion in *Amh* exon VI (*AmhΔy* [12]); a 547 bp band was present in nearly all sires and dams showed no band (Except 1D). **c** Exon 0 deletion (161 bp) in *AmhΔy* [12]; a 547 bp band was present in all individuals except 22S (purple circle, putative YY) and nearly all males showed a band with 386 bp (had 161 bp deletion). The other exceptions to the consensus patterns are: red circle indicates dam and sire concluded to be wrongly labelled (transposed) individuals, green circle indicates sire concluded to be an XX neo-male, progeny information was available for blue circled sire but the dam information was not available. M - molecular marker (100 bp), number = family number from Supplementary Table S1; D - dam, S – sire, DW - distilled water. Stirling broodstocks were non-informative

Analysis of the SNP (*ss831884014* [27]) in exon VI of *Amh* did not reveal any polymorphism in the GIFT fish and similar results were found following Sanger sequencing (Additional file 6: Data S2). However other assayable *Amh* polymorphisms (*Amh* exon VI, exon VII and *Amh*_E0) were found to be significantly associated with phenotypic sex in all the GIFT families and broodstock tested (Fig. 3 and Additional file 6: Data S2). In *AmhΔy*, a 233 bp deletion in exon VII, 5 bp insertion from *Amh* exon VI and 161 bp deletion in exon 0 were found to be nearly always associated with the male phenotype (Fig. 3a-c, Table 2 and Additional file 6: Data S2). These and the two microsatellites were always associated with the male phenotype in two GIFT families (no. 4, 7 and Additional file 6: Data S2), while in the other four families that were genotyped, there were 10–20% mismatches between LG23 markers (these markers and the two microsatellites) and sexual phenotype (Table 2). Families 4 and 7 showed 100% association, family 3 had 90% match, family 1 had 85% match, and families 2 and 19 had 80% match between the markers genotyped and the sexual phenotype (Table 2).

Five broodstock showed atypical genotypes for the two sex-linked microsatellites and *Amh* variants, given their phenotypic sex. The sires of families 20 and 27 appeared to be XX neomales (phenotypically male but genetically female) from these markers (lacked the male-associated alleles for both microsatellites, lacked the two male-associated deletions and one insertion in *AmhΔy*), while the dam of family 20 appeared to be normal XX female; the progeny of family 20 were nearly all (98.6%) females, supporting this interpretation, but the progeny sex-ratio of family 27 was not significantly different from 1:1 (dam of family 27 was missing). The sire of family 22 appeared to be a YY male (two copies of the male-associated alleles for both microsatellites, male-associated PCR bands only for insertion and deletion in *AmhΔy* exons VI and VII respectively), while the dam appeared to be a normal XX female; the progeny sex-ratio was highly skewed to males (89.9%). In family 1, the sire had a genotype typical of a female while the dam was the reverse, and the progeny sex-ratio was not significantly different from 1:1. This suggested that the parents had been wrongly labelled (i.e. male and female tissue samples transposed).

A 5608 bp promoter deletion in *Amhy* was also observed in the XY and putative YY GIFT males studied, with the larger allele (observed in XX individuals) presumably failing to amplify (*Amh*-linked band) in XY individuals due to preferential amplification of the smaller PCR product (Fig. 2c).

A three base pair insertion was also identified in exon 0 in *Amhy*. Male broodstock showed this 3 bp insertion (253 and 256 bp PCR products), while females did not (only 253 bp PCR product), with three exceptions out of the 50 broodstock analysed - two males and one female (Additional file 6: Data S2). Eleven GIFT sires and one dam were sequenced to test for the polymorphism (C/T) in exon II [12] and thought to have a critical role in male sex-determination in Nile tilapia, associated with the T allele. The T allele was not detected in the GIFT individuals studied - all had the base C in this position.

## Discussion

Given the mixed origins of the GIFT strain, it was assumed that there may be multiple loci involved in sex determination. In this study, we developed a powerful extension of BSA and ddRADseq by applying pre-extraction pooling of tissue samples to ddRADseq for the analysis and identification of sex-determining region(s) in GIFT families, followed by the verification of the identified region with different molecular marker analyses. This allowed us to examine multiple families efficiently. This is the first genomic analysis of sex determination in the GIFT strain.

The LG23 markers (5 SNPs, 2 microsatellites, 5 bp insertion in *Amh* exon VI, 233 bp deletion in *Amh* exon VII) that were screened showed strong association with phenotypic sex in the GIFT families and broodstock. Stirling broodstock (two females and two males) were non-informative for any of the LG23 SNP markers analysed (except for *ss2017360168*, both parents were heterozygous and no association was observed) and no male-associated microsatellite alleles or Y-linked bands (in LG23) were evident in those broodstock (Fig. 3 and Additional file 6: Data S2).

The *AmhΔy* insertion and deletions strongly associated with phenotypic sex in the GIFT families were the same as previously found in the Japanese and Israeli population of Nile tilapia [12]. In contrast, the variations in *Amhy* were different in GIFT from the Japanese population [12]. No substitution (C- > T) was found in *Amhy* exon II while a three base pair insertion in exon 0 region in *Amhy* was found to be linked to the male sex determination in GIFT (no information in the Japanese population [12]). The SNP in exon II was thought to have a critical role in the male sex determination in Nile tilapia derived from the Japanese strain [12]. The lack of this structure in the GIFT strain indicates that the functional role suggested for this substitution is unlikely, or at least is not general, for the Nile tilapia.

### Family-based association study using BSA-ddRADseq

The DNA pooling strategy was originally used with standard molecular techniques to identify markers linked to genes or genomic regions of interest [46, 47]. With the rapid advancement of next generation sequencing (NGS) technologies, BSA has been incorporated with different NGS platforms, given the potential of sequencing many individuals at minimum cost. There are possibilities for errors in using BSA, however, and these can be more pronounced when using BSA combined with NGS [30, 45, 48].

Tissue samples were pooled in the present study, which might lead to more variation in the representation of the genetic material from each individual. However, this strategy reduced the extraction cost, time and labour and allowed simultaneous analysing of hundreds of individuals from a single population. In prior publications the genetic material (DNA or also RNA/cDNA) was extracted individually followed by pooling of approximately equal amounts of nucleic acids. Pre-extraction pooling of tissue samples before DNA extraction for NGS has been applied on a limited scale in plants [48] and invertebrates [49] but no reports were found on the pooling of vertebrate animal tissue samples and the challenges of using this for NGS techniques.

Considering the variation likely to be present within the pooled tilapia progeny samples, only bi-allelic polymorphic loci showing Mendelian inheritance were used for the association analysis. In the two Stirling families a clear, strong association signal was identified between phenotypic sex and LG1 markers from the BSA-ddRAD analysis as expected, as the same association was found using known LG1 markers before constructing the BSA-ddRAD library and in a previous study with the same stock [24]. On the other hand, a strong association was found in a different chromosome (LG23) in the majority of the GIFT families.

Earlier BSA studies have reported different numbers of individuals being used to construct the pool for population genetics or genotype-phenotype association analyses. For example, BSA with NGS performs well when 50 individuals are pooled and larger pools than this (> 100) can result in even higher accuracy in allele frequency estimates [50], though this is dependent on sequencing effort (depth of coverage). Such large numbers are sometimes not feasible for some species, e.g. endangered ones. Another report has indicated that ≥50 individuals for haploid organisms or > 20 individuals for diploid organisms in a pool for NGS would have the power to estimate allele frequency accurately [51]. It has also been suggested that 10 to 20 individuals in each pool are sufficient to screen markers affecting a specific trait of interest, for example candidate gene mapping, QTL mapping and SNP marker discovery [52]. In the present study, 15 to 30 individuals were used in each pool, with an equal number of individuals of each sex to make the pools in each family. When pools were constructed with 30 individuals, some families showed very strong association of the SNPs to the phenotypic sex (Family 3, 4 and 13), some showed weaker association (Families 2 and 6) and some showed no association (Families 5 and 19). In case of pooled samples where 15 individuals were used, one family showed strong association (Family 7), one family showed weaker association (Family 10) and one family did not show any association (Family 14 and Additional file 1: Fig. S1). This suggests that the variation in the number of individuals per pool had little effect in the present study.

It has been noted for standard RADseq that increasing the sample size in a pool increases the occurrence of allelic skewing or dropout by increasing the chances of assaying individuals with mutations within the restriction site [53]. This type of problem is yet to be explored for ddRADseq where two restriction enzymes are used; it is likely to increase the probability of allelic dropout if the restriction enzyme cut sites are polymorphic. The power of the pooling strategy has been improved in some studies by making up multiple pools from the same individuals, replicating the pools for genotyping or sequencing, or increasing the sequencing read depth [54, 55]. In the current experiment, pooling had no apparent impact on the number of polymorphic filtered loci or the strength of association between the phenotypic sex and SNPs in the GIFT families studied.

### Identification and verification of the sex-determining region in GIFT

Even the cases of the families that did not show any association (Family 19) or showed a weaker association (Families 1, 2 and Additional file 1: Fig. S1) in the BSA-ddRADseq analysis, a significant association was found between phenotypic sex and all the markers in LG23 in the later analysis of individual samples for each family.

From individual analyses of the six GIFT families, departure of markers from the expected phenotypic sex was found to be common for the same individuals (Additional file 6: Data S2). It was found that in those families showing weaker or no association in BSA-ddRADseq analysis, more phenotypic males were found with the expected female genotype in the individual analysis of the six GIFT families (Table 2). In the case of other families which were not analysed individually, and which also had weaker or no association with LG23 markers, this could also be because of human error in the assessment of phenotypic sex. For example, four phenotypic males, based on microscopic sexing (two in Family 1, one in Family 2 and one in Family 19), had female genotypes but on visual external sexing those individuals had been identified as females. Similarly, another four phenotypic females based on microscopic sexing (one in Family 2, one in Family 3 and two in Family 19) were assessed as males from visual external sexing. Alternatively, the errors could arise from variation of representation of the genetic material in the pooled sample. Those factors, or others such as minor genetic or environmental factors, or the complex genetic structure of the GIFT strain, could alone or in combination influence the weaker genotype-phenotype associations from the BSA-ddRADseq analysis in some GIFT families.

Two major (XX/XY) sex-determining loci (in LG1 and LG23) have been found in Nile tilapia, and variants in the candidate gene complex (*Amh*) within the LG23 locus have been detected. There is no published evidence of the nature of the polymorphism acting as an XX/XY locus in LG1, and in particular no evidence of *Amh* or variants at this locus, suggesting that the sex-determining polymorphisms in LG1 and LG23 may be different. Temperature dependent sex-ratio is also evident in some strains of Nile tilapia and loci in LG1, 3, 20 and 23 show polymorphisms that have been linked with temperature effects on sex-ratio [25–27]. There are relatively few well-studied cases in fish taxa that can be compared to this. Three different strong candidate genes were found to be responsible for male sex determination in three closely related species of Medaka [5, 7, 8]. In contrast, a single gene (*sdY*) has been found to be the master sex-determining gene in all salmonids [13, 14], and it has been shown to have been transposed between different chromosomes during evolution, even being found in different chromosomal locations among individuals from a single aquaculture population of Atlantic salmon [56]. Given the evidence for polymorphism in XX/XY loci in the Nile tilapia, and the multiple origins of the GIFT strain, it was surprising to find that sex determination in the GIFT strain appears to be uniform: a single XX/XY locus in LG23, with no variation detected in the Y haplotype.

It has been reported that the members of the transforming growth factor beta (TGF-β) signalling pathway (*Gsdfy*, *Amhy* and *Amhr2*) could be part of a common pathway for sex determination in many fish [8–10]. Variations of the *Amh* gene (either *AmhΔy* or *Amhy*), a member of the TGF-β superfamily appear to be the candidate gene for male sex determination in GIFT.

## Conclusions

Pre-extraction pooling of tissue samples for BSA-ddRADseq proved to be an efficient alternative to individual sequencing or post-extraction pooling in family-based association studies. This allowed relatively rapid screening of multiple families in the GIFT strain, leading to mapping of a single sex-determining locus in LG23 and sex-linked SNP markers, with reduced experimental costs. This method could be used to map a range of other loci affecting important phenotypic traits using different NGS platforms.

This is the first genomic study of sex determination in the GIFT tilapia strain and only one locus (LG23) was identified as the major (XX/XY) sex-determining locus in GIFT across the population. No direct efforts were made to determine whether one of the Y-linked *Amh* variants in GIFT is actually the sex-determining gene, but the missense SNP in exon II of *Amhy*, proposed to be key in male determination [12], was absent in the GIFT individuals analysed in the present study. The tightly sex-linked LG23 markers in GIFT could be used in marker-assisted selection in GIFT to produce all-male populations for controlling sex-ratio in culture systems.

## Methods

### Sample collection, tissue preparation and genomic DNA extraction

Phenotypic sex data from twenty-eight GIFT families from generation 12 broodstock, produced by WorldFish Center (Penang, Malaysia), were made available to the project. From these, 19 GIFT families (parents and progeny) were selected (1–19 in Additional file 2: Table S1), and fin tissue from these, together with the remaining 12 broodstock (one sire and four dam were missing and one sire was used twice), were received and processed for further analysis. In addition, two families from the Stirling Nile tilapia population were included as positive controls for the BSA-ddRAD analysis, after first verifying that the phenotypic sex-ratio was balanced and strongly associated only with SNP markers in LG1 [24]. The two Stirling Nile tilapia families were produced in the Tropical Aquarium Facilities, Institute of Aquaculture. Phenotypic sex was determined by microscopic examination of gonad tissue, and fin clips fixed in 100% ethanol were used as the source of DNA. The phenotypic sex-ratios for the GIFT and Stirling families used in this study are given in Additional file 2: Table S1.

Rather than extract DNA from each progeny separately, a simpler, less time-consuming approach was taken for BSA. Fin tissue samples were pooled within each family according to progeny phenotypic sex. An equal number of each sex (at least 15 per sex) was used to make the two pools (male and female progeny) for each family (Table 3). A sterile 3 mm sized biopsy punch (Stiefel Laboratories Ltd) was used to take an approximately equal amount of fin tissue from each individual and half of this sample was added to the tissue pool for DNA extraction. The remaining half was retained for analysis of individual samples, if required.

**Table 3:** Number of individuals used to make DNA pools for each family for BSA-ddRAD library construction followed by number of individuals for each six GIFT families, and GIFT and Stirling broodstock for individual analysis with LG23-linked DNA markers

		BSA-ddRADseq analysis		LG23-linked marker analysis
ID	Strain	Individuals in male progeny pool	Individuals in female progeny pool	Individuals analysed
Family 1	Stirling	24	24	
Family 2	Stirling	29	29	
Family 1	GIFT	25	25	40
Family 2	GIFT	30	30	40
Family 3	GIFT	30	30	40
Family 4	GIFT	30	30	40
Family 5	GIFT	30	30	
Family 6	GIFT	30	30	
Family 7	GIFT	15	15	30
Family 8	GIFT	18	18	
Family 9	GIFT	28	28	
Family 10	GIFT	15	15	
Family 11	GIFT	21	21	
Family 12	GIFT	22	22	
Family 13	GIFT	30	30	
Family 14	GIFT	15	15	
Family 15	GIFT	22	22	
Family 16	GIFT	17	17	
Family 17	GIFT	23	23	
Family 18	GIFT	23	23	
Family 19	GIFT	30	30	40
Broodstock	GIFT			50
Broodstock	Stirling			4

Genomic DNA from individual samples was extracted using a salt precipitation method known to yield very high molecular weight DNA from tilapia species [57]. Briefly, individual fin tissue from parents (c. 0.25 cm^2^) were digested in lysis solution (220 μL SSTNE containing 1% SDS and 100 μg proteinase K). The SSTNE buffer (pH 9) comprised 50 mM Tris base, 300 mM NaCl, 0.2 mM each of EGTA and EDTA, 0.15 mM of spermine tetrahydrochloride, and 0.28 mM of spermidine trihydrochloride. Following overnight digestion at 55 °C, 5 μL RNaseA (2 mg/mL) was added and samples were incubated at 37 °C for a further 60 min to degrade RNA. Proteins were precipitated by the addition of 0.7 volumes 5 M NaCl, these being pelleted by centrifugation. DNA was precipitated from the isolated supernatant by addition of 0.7 volumes isopropanol and pelleted by centrifugation. Following two 70% ethanol washes over a 14-h period, the DNA pellet was dissolved in c. 30 μL of 5 mM Tris (pH 8.0). Solutions were proportionately scaled up for DNA extraction for the pooled progeny samples. Genomic DNA quantification, purity and integrity were assessed using spectrophotometry (Nanodrop, Labtech International Ltd) and agarose gel electrophoresis. For library construction double-stranded DNA (dsDNA) was measured more accurately by QUBIT fluorimetry and was diluted to a standard concentration of 8 ng/μL with 5 mM Tris (pH 8.5).

### BSA-ddRADseq library preparation

Two BSA-ddRAD libraries were prepared, sequenced and analysed sequentially. The first library was constructed for five GIFT and two Stirling families as a pilot run. Based on the results from this sequencing it was gauged that a single MiSeq run for a second library would provide sufficient sequence coverage for the remaining 14 GIFT families. The samples in the first library were replicated four times, i.e. four separate restriction enzyme digestions/ligations/individual barcodes, whereas samples were not replicated in the second library. Each family comprised four DNA samples, i.e. from dam, sire, male progeny pool and female progeny pool. The BSA-ddRAD libraries were prepared using a modified version of the original ddRAD methodology [31] described in detail elsewhere [35, 58]. Briefly each sample (24 ng DNA) was digested with *Sbf*I (CCTGCA^GG) and *Sph*I (GCATG^C) high fidelity restriction enzymes (New England Biolabs, UK) at 37 °C for 90 min using 20 U of restriction enzyme per μg of genomic DNA in 10× CutSmart reaction buffer (New England Biolabs, NEB). Each digested DNA sample was then ligated with individual-specific P1 (*Sbf*I compatible) and P2 adapters (*Sph*I compatible) for 2.5 h at 22 °C, each with a unique 5 or 7 bp barcode (see barcode information in Additional file 7: Data S3). Ligation was stopped by adding 2.5 volumes PB buffer (Qiagen, UK) and all samples were multiplexed into a single library pool and purified with a single column (MinElute PCR purification kit, Qiagen, UK). Fragments were then size selected on a 1.1% agarose gel with a portion corresponding to c. 400–700 bp being excised and gel purified (MinElute gel purification kit, Qiagen UK). This template was subjected to 11 cycles of PCR (using Q5 Hot-start High Fidelity DNA polymerase (NEB) and Illumina specific primers) and the amplified library was purified twice; first by a column purification (MinElute PCR purification kit) then by a paramagnetic bead clean up (AMPure XP, Beckman Coulter, UK). The final library was sequenced on the Illumina MiSeq platform (v2 chemistry, 161 base paired-end reads).

### Computational methods for generating RAD loci

Raw sequence data were processed through the FASTQC software (Version 0.11.2) to check initial quality of the sequencing runs. Sample reads were demultiplexed by the *process_radtags.pl* component in STACKS (version 1.27) [59], using the default parameters except the quality score filter (−s) was increased from 10 to 20. Low quality reads, reads missing restriction enzyme cut sites and reads with ambiguous or unpaired barcodes were filtered out during demultiplexing. Filtered reads were aligned to the published Nile tilapia genome (genome assembly Orenil1.1) [60] using the default parameters of the Bowtie2 aligner (version 2.2.9) [61]. Reads were then sorted into loci using the default parameters of *ref_map.pl* component in the STACKS. The major STACKS parameters implemented were: the minimum depth of coverage to build a stack (−m) of 6 and the mismatches allowed between catalogue loci (−n) of 2.

### Genome-wide association studies to identify the sex-determining region

Following the reference-based assembly within STACKS, a custom Perl script was used to filter the data to extract informative, robust loci prior to downstream analysis. The filtering for each family dataset comprised: 1) monomorphic RAD loci were removed; 2) RAD loci with more than two SNPs were discarded; 3) only RAD loci common to dam, sire, male progeny pool and female progeny pool were retained; 4) only bi-allelic loci for parents were included; 5) the presence of both parental alleles in either (or both) progeny pools was ensured.

Following this filtering, a Fisher’s exact test was performed between the datasets from the two progeny pools, using the exact nucleotide/allelic counts for each SNP. The corrected *p*-values (*q*-value) were calculated using the R/qvalue package, a package that implements a false discovery rate (FDR) method for genome-wide tests of significance. To identify the positional candidate SNPs linked to sex for each family, *q*-values were visualised according to the physical position in the Nile tilapia genome, using Manhattan plots in the R/qqman package [62] (Additional file 1: Fig. S1). The *q*-values from all the families (two and 19 families in the Stirling and GIFT families respectively) were combined together and were visualised according to the physical position in the Nile tilapia genome following the same programme described above (Fig. 1).

### Analysis of sex-linked markers in LG23

The two Stirling families (*n* = 110) were analysed individually using tightly sex-linked SNP (*Oni23063* and *Oni28137*) and microsatellite (*UNH995*) markers in LG1 [24] and a SNP (*Oni3161*) in LG20 associated with thermosensitivity in this population [25] to confirm the genotype-phenotype association before constructing the BSA-ddRAD library. The five most highly significantly sex-associated SNPs from BSA-ddRADseq analysis in GIFT and two microsatellite markers (*UNH898*, *ARO172*) tightly linked to sex in LG23 [28] were selected and analysed for progenies of six GIFT families (based on the high, low and not significant association between the LG23 marker and phenotypic sex from BSA-ddRADseq analysis), and fifty GIFT and four Stirling broodstock (Table 3; NCBI dbSNP accession *ss2017360134*, *ss2017360168*, *ss2017360173*, *ss2017360175* and *ss2017360178*). A missense SNP (*ss831884014*) in exon VI of *Amh* was also tested for fifty GIFT and four Stirling broodstock [27] and a SNP in LG23 (*ss2017360168*) for two Stirling families was also analysed. RAD-tag sequences and primer sequences for allele-specific primers and microsatellite markers are provided in Additional files 8-10: Data S4-S6.

SNPs were genotyped using fluorescence-based Kompetitive Allele Specific end-point PCR (KASP) genotyping system (KBioscience UK Ltd) following a previously published protocol [63, 64]. The assay volume was 5 μL (c. 25 ng DNA) and the PCR was performed using the following cyclic conditions: the initial denaturation at 94 °C for 15 min followed by 10 touchdown cycles (94 °C for 20 s and touchdown 65 °C for 1 min, reduced by 0.8 °C per cycle) followed by 34 cycles of amplification (94 °C for 20 s; 57 °C for 1 min). Microsatellite markers were analysed using a fluorescent labelled tailed primer method [64, 65]. In brief, 5 μL (c. 25 ng DNA) PCR reaction volumes were prepared and the thermocycling conditions were the initial denaturation at 95 °C for 1 min and 35 cycles of denaturation at 95 °C for 15 s, annealing at 62 °C for 15 s and extension at 72 °C for 30 s.

The recently identified Y-linked *Amh* gene variants [11, 12] were also analysed. The insertion in *Amh* exon VI and deletion in *Amh* exon VII were screened in progenies of six families, and fifty GIFT and four Stirling broodstock (*n* = 284, Table 3) using a standard PCR protocol and the amplified products (3 μL) were checked on 1.5% agarose electrophoresis. The PCR was carried out in 5 μL reaction volumes (c. 25 ng DNA) and the thermocycling conditions were initial denaturation at 95 °C for 1 min and 35 cycles of denaturation at 95 °C for 15 s, annealing at 62 °C for *Amh* exon VII (whereas 67 °C for 5 bp insertion in *Amh* exon VI) for 15 s and extension at 72 °C for 30 s. The promoter deletion (5608 bp) in *Amhy* [12] (*Amhy*_Promoter_del, Additional file 10: Data S6) was checked for five GIFT individuals (one female and four males). PCR was performed with TaKaRa LA Taq® Hot Start DNA polymerase and the cyclic conditions were the initial denaturation at 96 °C for 2 min, 30 cycles of denaturation at 96 °C for 40 s, annealing at 63 °C for 30 s and extension at 72 °C for 8.5 min, with the final extension at 72 °C for 10 min. The exon 0 deletion in *AmhΔy* (*Amh*_E0) was analysed for fifty GIFT and four Stirling broodstock. The primer sequences are in Additional file 10: Data S6.

Following screening of the SNP in *Amh* exon VI for fifty GIFT and four Stirling broodstock using KASP (see above), six broodstock (two dams and two sires from GIFT, and the dam and sire for Stirling family 1) were screened again for this SNP (*Amh*_SNP_exon_VI, primers were designed flanking the SNP [27]) using Sanger sequencing, following the manufacturer’s protocols (GATC Biotech; Sanger ABI 3730 × l, LIGHTRUN™ sequencing service). Similarly, for the SNP in *Amhy* exon II, eleven randomly chosen GIFT sires and one dam were analysed using Sanger sequencing (*Amhy*_E0_E2, forward primer was designed within the *Amhy* E0 region and the reverse primer was designed within the *Amhy* intron II). A 3 bp insertion in *Amhy* exon 0 (*Amh*_E0_del, primers were designed from upstream of *Amh* exon 0 to downstream of exon 0) was analysed for fifty GIFT and four Stirling broodstock. PCR products were run on a CEQ genotyping machine for fragment analysis. Primer sequences for all the markers tested are provided in Additional file 10: Data S6.

### Association analysis between DNA markers and phenotypic sex in GIFT

An association analysis between genotype and phenotypic sex for each LG23 SNP marker was conducted for each family and broodstock using the SNPassoc package in R (version 3.1.3). A generalised linear model was applied under the function *WGassociation* to test the magnitude of association between each SNP marker and phenotypic sex. Significant *p*-values were corrected for multiple tests using the Bonferroni correction method. Fisher’s exact test was used to test for significance for association of microsatellite and *Amh* gene variations (located on chromosome Y) to the phenotypic sex.

## Supplementary information

**Additional file 1 Figure S1.** Genome-wide association plot with the phenotypic sex for each Stirling and GIFT family from BSA-ddRAD analysis. Each dot represents a SNP and the Y-axis represents the magnitude of association (−log10*P* value of F-test) of the SNP with phenotypic sex, while the X-axis represents the position in the linkage groups of the assembled Nile tilapia genome. The alternating blue and green colours are used to distinguish between chromosomes. The red solid line represents a *q*-value of 0.05 and the blue solid line represents a *q*-value of 0.01.

**Additional file 2 Table S1.** Progeny sex ratios with the Chi-square *p*-value (compared to a 1:1 sex ratio) for the individual GIFT and Stirling families used in this study.

**Additional file 3 Table S2.** Details of the number of sequencing reads before and after initial filtering, the number of ddRAD loci generated using the Nile tilapia genome as a reference and the final numbers of filtered polymorphic loci used for downstream analysis.

**Additional file 4 Table S3.** Association analysis of microsatellites and markers in the variations of the *Amh* gene on the Y chromosome with the phenotypic sex of progeny for each GIFT family separately and broodstock. Data shown are probabilities of association (see text for further details).

**Additional file 5 Data S1.** Genotype data for the two Stirling families for LG1 markers (*Oni23063*, *Oni28137* and *UNH995*), and LG20 marker (*Oni3161*) with their phenotypic sex.

**Additional file 6 Data S2.** Phenotypic sex and genotype information for each marker studied for six GIFT families, fifty GIFT and four Stirling broodstock.

**Additional file 7 Data S3.** Details of each sample used for BSA-ddRADseq: sample ID, sex, source of the sample, ddRADseq run, P1 and P2 barcode information and the generated paired-end reads.

**Additional file 8 Data S4.** KASP assay sequences: details of SNP alleles and RAD-tag sequences of the five markers.

**Additional file 9 Data S5.** KASP assay primer sequences: list of the allele-specific primers and common primer designed for the allele-specific PCR genotyping assay of the markers.

**Additional file 10 Data S6.** Primer sequences used in this study.

## Data Availability

The raw sequence data for this study were submitted to the EBI’s European Nucleotide Archive (ENA) Sequence Read Archive (SRA), study accession number PRJEB13792. Five SNPs were submitted to dbSNP NCBI and the accession numbers are *ss2017360134*, *ss2017360168*, *ss2017360173*, *ss2017360175* and *ss2017360178*.

## Abbreviations

BSA: Bulk segregant analysis
BSA-ddRADseq: Double-digest restriction-site associated DNA sequencing
dsDNA: Double-stranded DNA
ENA: European nucleotide archive
FDR: False discovery rate
GBS: Genotyping-by-sequencing
GIFT: Genetically improved farmed tilapia
LG: Linkage group
MAS: Marker-assisted selection
NGS: Next generation sequencing
SNP: Single nucleotide polymorphism
SRA: Sequence read archive
SSR: Simple sequence repeats
TGF-β: Transforming growth factor beta
QTL: Quantitative trait locus
