# Supplementary material for: Sex determination in the GIFT strain of tilapia is controlled by a locus in linkage group 23
Source: BMC Genet. 2020 Apr 29;21:49. doi: 10.1186/s12863-020-00853-3 (PMC7189693; doi:10.1186/s12863-020-00853-3)

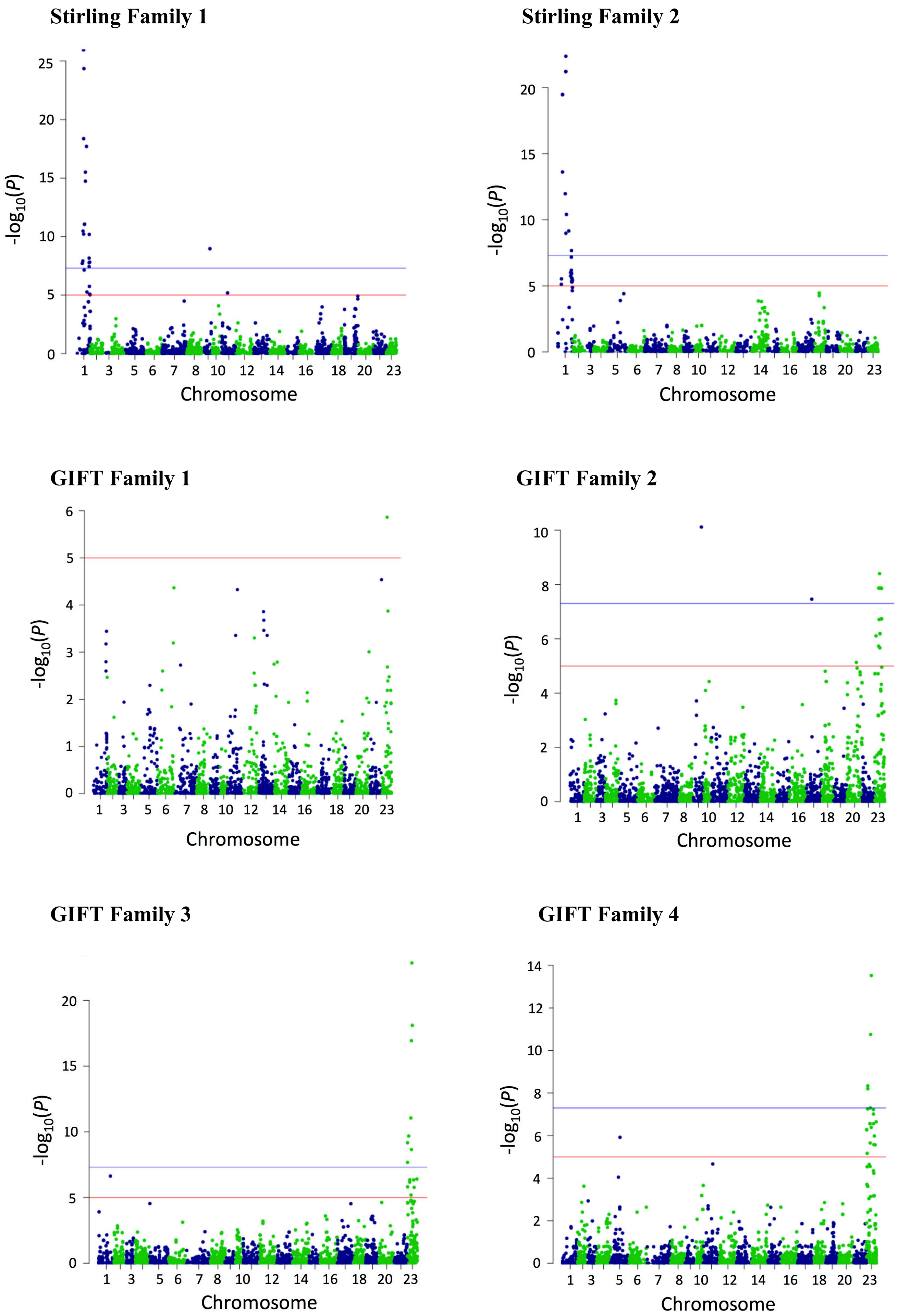

Stirling Family 1
Stirling Family 2
GIFT Family 1
GIFT Family 2
GIFT Family 3
GIFT Family 4
$-\log_{10}(P)$
Chromosome

GIFT Family 5

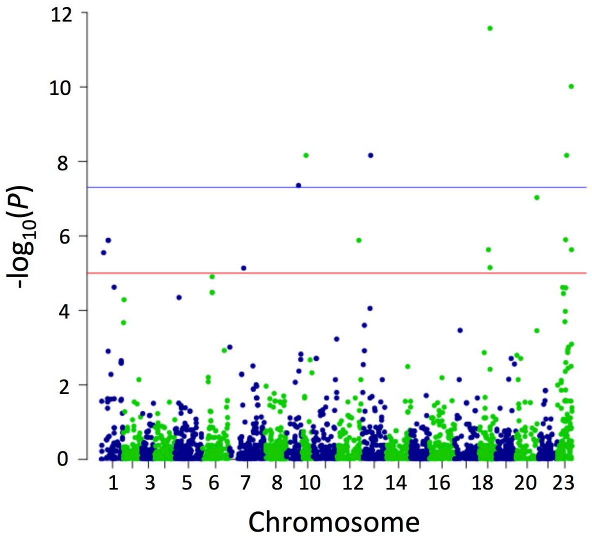

GIFT Family 6

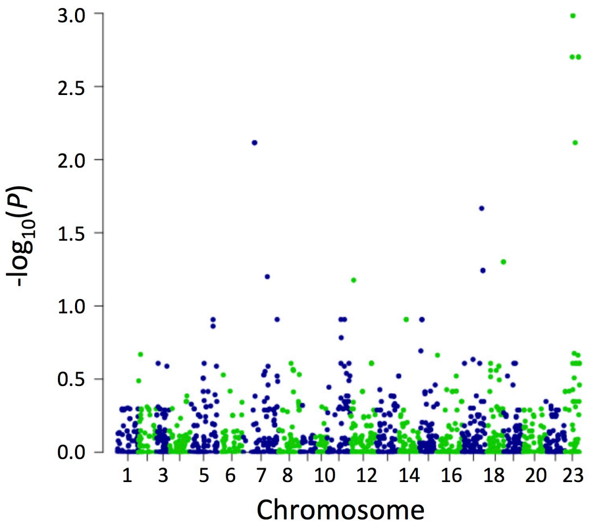

GIFT Family 7

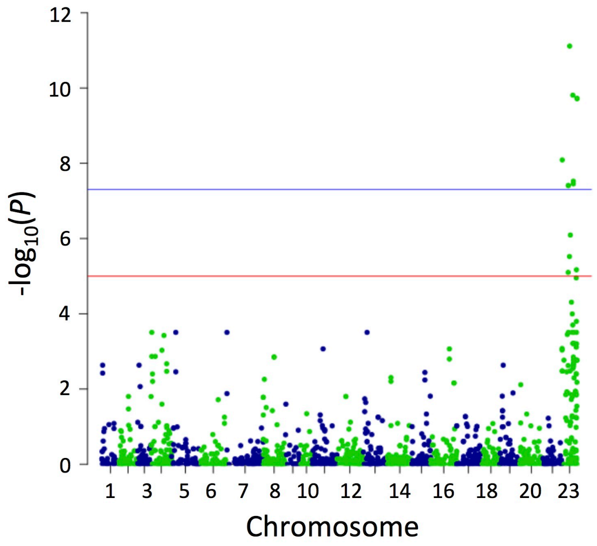

GIFT Family 8

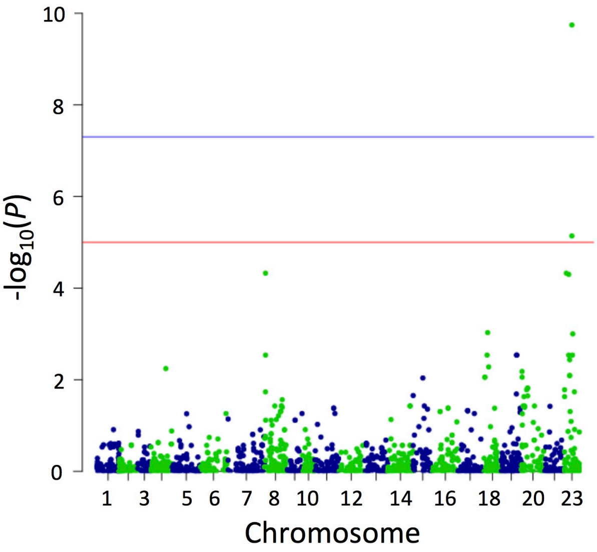

GIFT Family 9

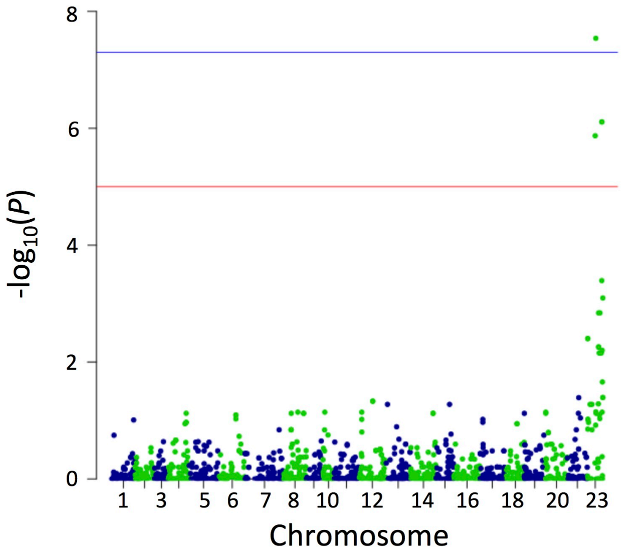

GIFT Family 10

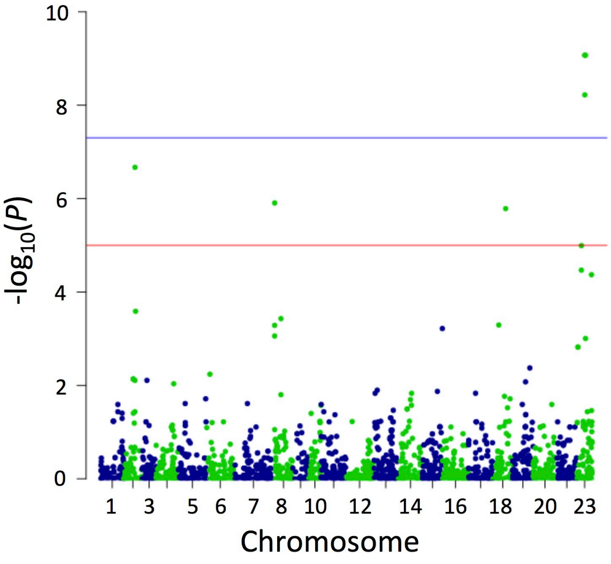

GIFT Family 11

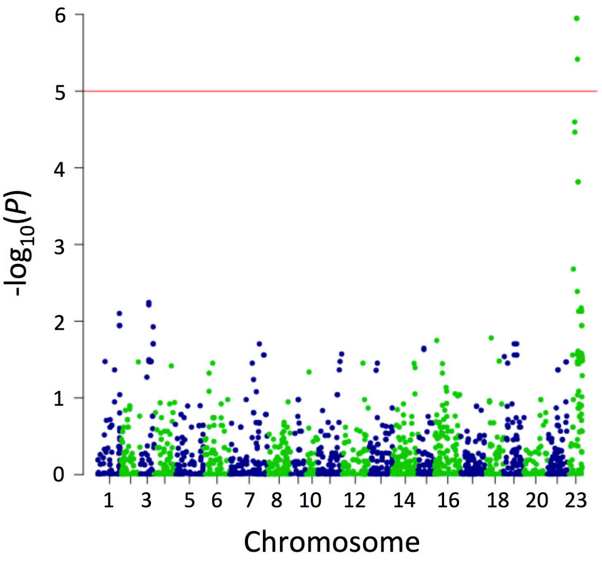

GIFT Family 12

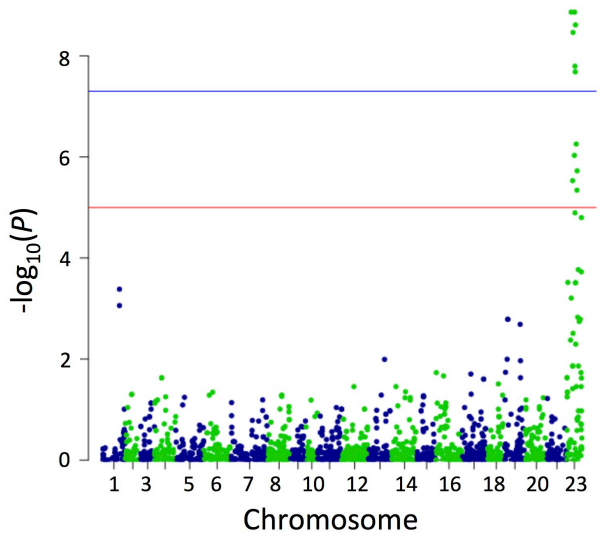

GIFT Family 13

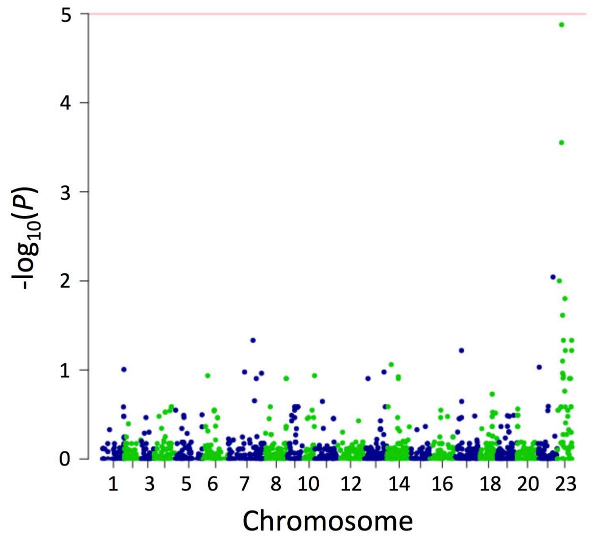

GIFT Family 14

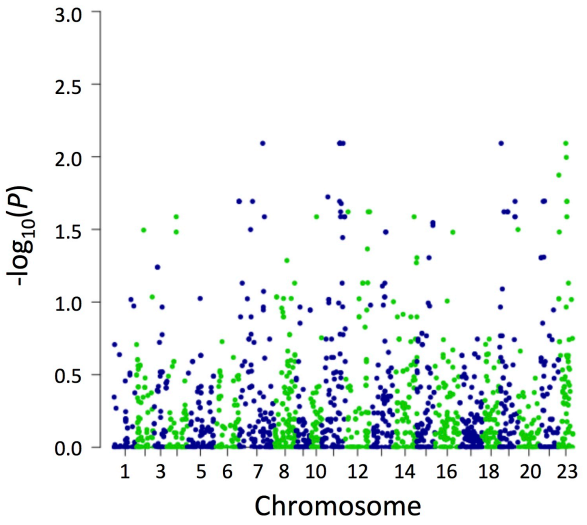

GIFT Family 15

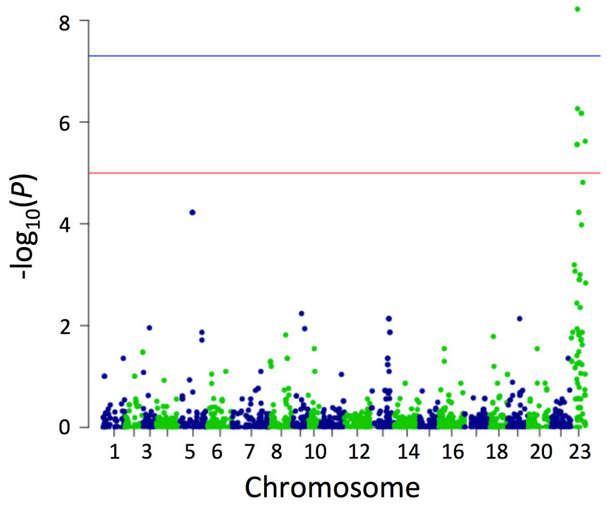

GIFT Family 16

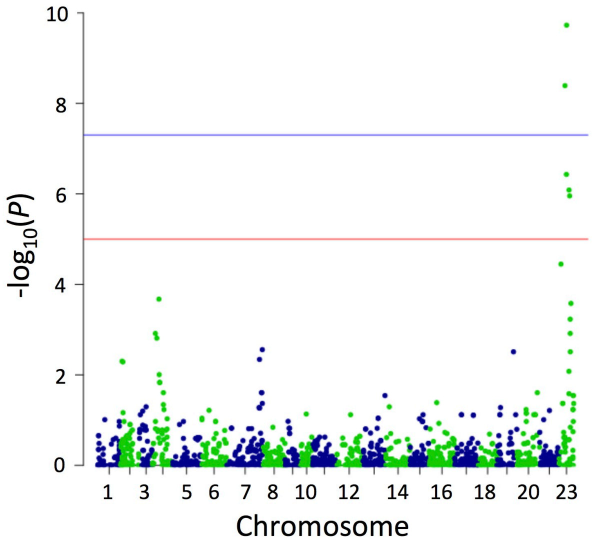

GIFT Family 17

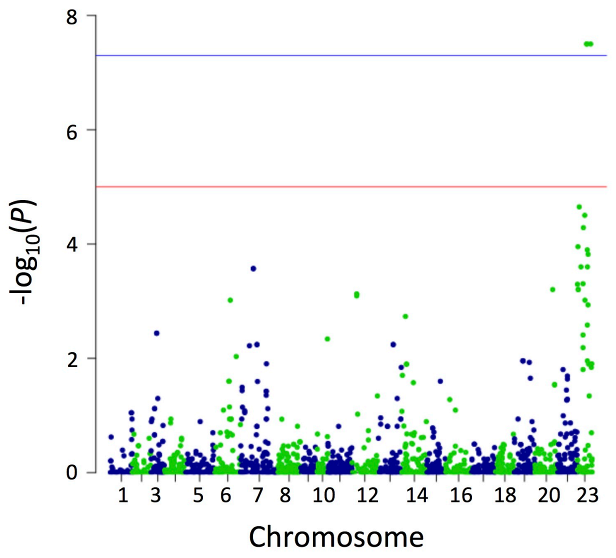

GIFT Family 18

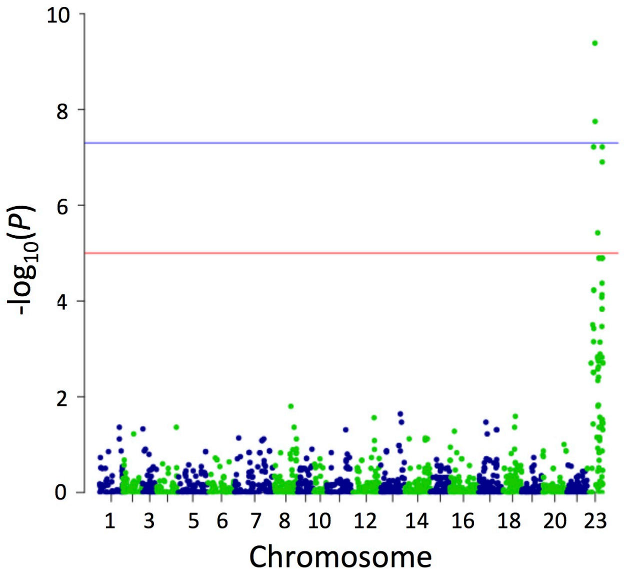

GIFT Family 19

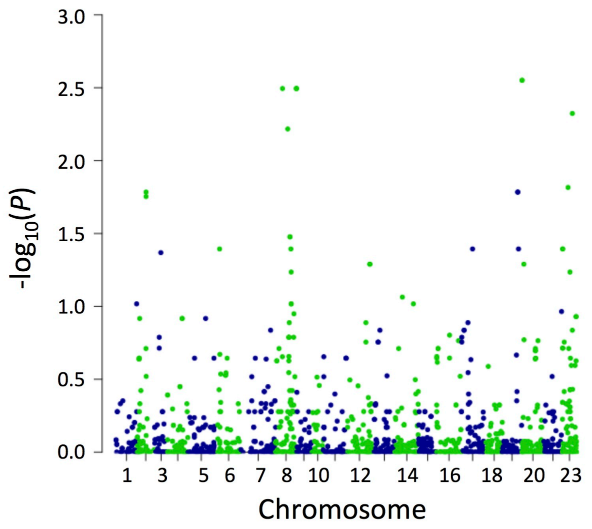

Supplement: Supplementary file 1 — Additional file 1 Figure S1. Genome-wide association plot with the phenotypic sex for each Stirling and GIFT family from BSA-ddRAD analysis. Each dot represents a SNP and the Y-axis represents the magnitude of association (−log10P value of F-test) of the SNP with phenotypic sex, while the X-axis represents the position in the linkage groups of the assembled Nile tilapia genome. The alternating blue and green colours are used to distinguish between chromosomes. The red solid line represents a q-value of 0.05 and the blue solid line represents a q-value of 0.01. [file 12863_2020_853_MOESM1_ESM.pdf]
